# Measuring anxiety after spinal cord injury: Development and psychometric characteristics of the SCI-QOL Anxiety item bank and linkage with GAD-7

**DOI:** 10.1179/2045772315Y.0000000029

**Published:** 2015-05

**Authors:** Pamela A. Kisala, David S. Tulsky, Claire Z. Kalpakjian, Allen W. Heinemann, Ryan T. Pohlig, Adam Carle, Seung W. Choi

**Affiliations:** 1 Department of Physical Therapy, University of Delaware, College of Health Sciences, Newark, DE, USA; 2 Kessler Foundation Research Center, West Orange, NJ, USA; 3 Department of Physical Medicine and Rehabilitation, University of Michigan Medical School, Ann Arbor, MI, USA; 4 Rehabilitation Institute of Chicago, and Department of Physical Medicine and Rehabilitation, Feinberg School of Medicine, Northwestern University, Chicago, IL, USA; 5 Cincinnati Children's Hospital, Cincinnati, OH, USA; 6 McGraw–Hill Education CTB, Monterey, CA, USA

**Keywords:** Health-Related Quality of Life, Outcomes Assessment (Healthcare), Patient Reported Outcomes, Anxiety, Spinal Cord Injuries

## Abstract

**Objective:**

To develop a calibrated item bank and computer adaptive test to assess anxiety symptoms in individuals with spinal cord injury (SCI), transform scores to the Patient Reported Outcomes Measurement Information System (PROMIS) metric, and create a statistical linkage with the Generalized Anxiety Disorder (GAD)-7, a widely used anxiety measure.

**Design:**

Grounded-theory based qualitative item development methods; large-scale item calibration field testing; confirmatory factor analysis; graded response model item response theory analyses; statistical linking techniques to transform scores to a PROMIS metric; and linkage with the GAD-7.

**Setting:**

Five SCI Model System centers and one Department of Veterans Affairs medical center in the United States.

**Participants:**

Adults with traumatic SCI.

**Main Outcome Measures:**

Spinal Cord Injury-Quality of Life (SCI-QOL) Anxiety Item Bank

**Results:**

Seven hundred sixteen individuals with traumatic SCI completed 38 items assessing anxiety, 17 of which were PROMIS items. After 13 items (including 2 PROMIS items) were removed, factor analyses confirmed unidimensionality. Item response theory analyses were used to estimate slopes and thresholds for the final 25 items (15 from PROMIS). The observed Pearson correlation between the SCI-QOL Anxiety and GAD-7 scores was 0.67.

**Conclusions:**

The SCI-QOL Anxiety item bank demonstrates excellent psychometric properties and is available as a computer adaptive test or short form for research and clinical applications. SCI-QOL Anxiety scores have been transformed to the PROMIS metric and we provide a method to link SCI-QOL Anxiety scores with those of the GAD-7.

## Introduction

Generalized anxiety disorder (GAD) was introduced into the Diagnostic and Statistical Manual of Mental Disorders, 3rd Edition^1^ in 1980, and reflects the evolution of the definition and diagnosis of anxiety as an entity distinct from panic.^2^ Current diagnostic criteria are available in the DSM-5^3^ and continue to reflect excessive levels of anxiety and worry and exhibits a high degree of comorbidity with other anxiety and mood disorders.^2^ Even in the absence of comorbid mental health disorders, anxiety in its most uncomplicated form is associated with a significant economic and personal burden, including a high use of health care services and reduced productivity.^4^ Despite the pervasive and adverse impact of GAD and the greater use of health services, few individuals seek treatment.^2^

It is important to note that an elevated level of anxiety is a component of many mental disorders and is not necessarily an indicator of GAD. For traumatic events such as spinal cord injury (SCI), individuals may experience a wide range of anxious symptoms. Some may have little or slightly elevated anxiety while, for others, anxiety may be a symptom of another disorder, such as post-traumatic stress disorder or adjustment disorder.^1^ As is the case in the broader mental health literature, little attention has been paid to anxiety in individuals with spinal cord injury (SCI), despite early recognition of anxiety as a sequela of injury. In 1979, Cook^5^ lamented the paucity of research addressing anxiety in persons with SCI; this oversight persists nearly 40 years later. The literature on anxiety after SCI is limited by small sample sizes, lack of controls, heterogeneity of samples, limited studies during the acute phase of recovery, diversity of measurement, lack of theory, lack of replication, exclusive focus on point-prevalence, ignoring lifetime prevalence or incidence, and few treatment trials.^6^

Early investigations of anxiety following SCI found the prevalence to be within general population ranges.^5,7^ Hancock *et al*. examined anxiety and depression in the first year post-injury. They found the prevalence of anxiety and depression were significantly higher in persons with SCI compared to controls, though mean scores were in the mild range of severity.^8^ Craig *et al.* found no significant improvement in anxiety or depression in the second compared to the first year post-SCI; about 30% of the sample had elevated scores.^9^ Bonanno *et al.* reported subsets of a SCI sample with stable/low, delayed, and improved trajectories of anxiety improvement.^10^ In a cross-sectional sample, anxiety was related to level and completeness of injury, pain, motor and sensory loss, bowel dysfunction, and shorter duration between injury and rehabilitation.^6^

Screening measures of anxiety are typically embedded in scales that assess both anxiety and depression symptoms. In a recent review of anxiety and depression measures in SCI,^11^ no stand-alone measure of anxiety was identified. Rather, instruments such as the Depression Anxiety Stress Scales-21,^12^ Hospital Anxiety and Depression Scale,^13^ Ilfeld Psychiatric Symptom Inventory,^14^ and the Symptoms Checklist-90-Revised Research Subscales^15^ are used to assess anxiety. These scales have been criticized for complex loading of somatic items for both depression and anxiety^16^ and low specificity for detecting anxiety and depression.^17^

The Patient Reported Outcomes Measurement Information System (PROMIS)^18,19^ addresses limitations of earlier instruments. PROMIS contains an anxiety item bank^20^ which was developed with patient feedback to assure a wide range of items across the spectrum of anxiety from low to high severity symptoms. The anxiety items were calibrated using a graded-response item response theory (IRT) model^21^ with a large sample drawn from the general population.^20^

There are several advantages to IRT-based item banks over classical test theory-derived measures. In contrast to classical test theory methods that favor items of average symptom severity, IRT-developed scales contain items with a wide range of symptom severity. Including a broad range of items results in a bank that has greater reliability and measurement accuracy across a wider range of symptoms than classical test theory-based scores.^22^ IRT methods produce unidimensional item banks that can be used with computer adaptive testing (CAT); items can be administered quickly with a small number of items while maintaining precision. Anxiety measures that are sensitive to change and developed in a patient-centered manner are essential to monitoring treatment.

The purpose of this project was to develop an anxiety item bank, estimate model fit, calibrate items, and produce, a CAT and short form. We hypothesized that, with a few additions, the PROMIS Anxiety items would largely be suitable for an SCI population, and that there would be a moderate to strong correlation between the new SCI-QOL Anxiety bank and the GAD-7,^23^ a self-report measure of anxiety that is commonly used in SCI research. This manuscript describes the development, calibration, and psychometric characteristics of the SCI-QOL Anxiety item bank. We report scores using PROMIS’ general population metric and develop a crosswalk (i.e. statistical linkage) with the GAD-7, a widely used anxiety measure.

## Methods

### Development of an anxiety item pool

We began by identifying candidate items, which included semi-structured interviews and focus groups with patients with SCI and clinicians who specialize in SCI (see Tulsky *et al.*^24^ for a full description). We developed a set of 47 anxiety items based on the data from individual interviews. Then, specific phrases or concepts were drawn from the focus group transcripts and converted into 63 additional “new” anxiety items. For example, a focus group participant with complete tetraplegia talked about feeling “*trapped*”; like a “*prisoner in your own body*.” From these statements, we drafted the item “*I felt trapped in my body*”. We selected 27 additional items from the Neurology and Quality of Life (Neuro-QOL) measurement system (including 17 verbatim PROMIS items). In cases where newly written (i.e. based on interview or focus group feedback) items were redundant with these existing items, the new items were dropped in favor of the established Neuro-QOL/PROMIS items.

A total of 73 unique Anxiety items (including the 27 Neuro-QOL items/17 PROMIS items) proceeded through Expert Item Review (EIR),^25^ a method whereby project investigators considered items’ relevance and clarity, and suggested revisions and deletions. Based on EIR feedback, we retained 49 items. Team members reviewed and modified these items. We arranged items on a symptom severity hierarchy and subsequently removed 4 redundant items where there was oversaturation in the middle range of the hierarchy. Additionally, during this phase of review, the project investigators decided to develop a separate pool of items related to psychological trauma,^26^ and 7 of the more extreme anxiety items were moved to this new set of items. No new items were added at this time.

With the exception of the 27 Neuro-QOL/17 PROMIS items which had undergone cognitive debriefing,^27^ we asked individuals with SCI to answer each item, then describe the process they used to generate with their answer, and to identify confusing, unclear, or derogatory wording. No items were modified or deleted based on cognitive interviewing. After this phase, the remaining 38 items were reviewed for translatability (for method, please see Eremenco *et al.*)^28^ and reading level (using the Lexile framework).^29^ Slight modifications were made to 2 items after the translatability and cultural review. For example, the item “I felt trapped in my body” was changed to “I felt trapped in my *own* body,” since the phrase “in my body” is an idiomatic expression which would lose clarity upon translation. We wrote items no higher than a fifth grade reading level.

### Calibration study and GAD crosswalk participants and data collection procedures

The Institutional Review Board at each collaborating site reviewed and approved this project. We administered 38 anxiety items along with other item pools reflecting different health related quality of life (HRQOL) subdomains to persons with SCI as part of a multisite item calibration study. Participating sites included the Kessler Foundation, University of Michigan, Rehabilitation Institute of Chicago, University of Washington, Craig Hospital and the James J. Peters/Bronx Department of Veterans Affairs (VA) hospital.

Inclusion criteria were 18 years of age and older, ability to read and understand English, and medically-documented traumatic SCI. There were no additional exclusion criteria. We stratified the sample by level of injury (paraplegia vs. tetraplegia), completeness of injury (complete vs. incomplete), and time since injury (<1 year, 1–3 years, and >3 years) to ensure that the sample was heterogeneous. Stratification was achieved by targeted recruitment of each subgroup (e.g. paraplegia, complete, <1 year); we closely monitored the recruitment process and when an enrollment target for a subgroup cell was met, we prioritized recruitment of individuals who fit a different subgroup cell and no further individuals with those specific injury characteristics were enrolled. We confirmed diagnoses by medical record review; neurologic level was documented by the most recent American Spinal Injury Association Impairment Scale (AIS) rating.^30^ To meet the sample size requirements of the graded response model, the goal was to recruit a sample of at least 500 individuals with sufficient heterogeneity to ensure at least 5 participants chose each of the 5 responses for each item.

Interviewers administered items using a structured protocol in person or by telephone. Tulsky *et al*.^31^ describe the methods in detail. A subset of the sample also completed the GAD-7.

### Reliability samples and data collection procedures

Test-retest reliability was assessed with a separate sample of individuals with SCI at 4 Spinal Cord Injury Model System (SCIMS) centers (University of Michigan, Kessler Institute for Rehabilitation, Rehabilitation Institute of Chicago, and Craig Hospital). Participants were community dwelling individuals with traumatic SCI (>4 months post injury) who completed the SCI-QOL Anxiety CAT at baseline and 1-2 weeks as part of a larger study. All data were collected during a structured interview; the interviewer presented each CAT item as it appeared and entered all responses directly into the Assessment Center^SM^ platform.

### Calibration analysis

Data analysis included confirmatory factor analysis (CFA) to confirm construct unidimensionality, use of a graded-response IRT model to calibrate items, and examination of differential item functioning (DIF). For CFA, we defined good model fit as Comparative Fit Index (CFI) >0.90 and root mean square error of approximation (RMSEA)<0.08, and excellent fit as CFI > 0.95 and RMSEA < 0.06. We iteratively identified poorly fitting items by examining item fit to a graded response IRT model, DIF, local item dependence (LID) (residual correlations >|0.20|), and significant loadings on the single factor (values >0.30). We removed poorly fitting items and repeated the analytic steps. Once misfitting and DIF items were removed, we programmed a CAT on the Assessment Center website (www.assessmentcenter.net) and used item parameters and clinical input to select items for a brief, fixed-length form (“short form”).

### Reliability analysis

We calculated Pearson's *r* between baseline and 1–2 week assessments to assess test-retest reliability.

### Transformation to PROMIS metric

We report SCI-QOL Anxiety scores based on PROMIS normative data in which T-scores of 50 represent the mean of the general population rather than the mean for persons with SCI.^31^ We identified anchor items, those common to PROMIS and SCI-QOL, and used the Stocking-Lord method^32^ to link the measures. We examined item-response plots and scatter plots of item parameters, estimated transformation constants, and modified the initial item parameters accordingly.

### Crosswalk to GAD-7

We created a crosswalk between the SCI-QOL Anxiety item bank and the GAD-7 using PROsetta Stone procedures.^33,34^

## Results

### Participant characteristics of samples

Table 1 summarizes the demographic and injury characteristics of the calibration sample and GAD-7 subsample.

**Table 1:** Demographic and Injury Characteristics of Calibration Sample and GAD Crosswalk Subsample

Variable	Calibration Sample (*n* = 716) Mean (SD), *n* (%)	GAD Crosswalk Subsample (*n* = 465) Mean (SD), *n* (%)
Age (years)	43.0 (15.3)	41.8 (15.6)
Sex		
Male	558 (78%)	363 (78%)
Female	158 (22%)	102 (22%)
Ethnicity		
Hispanic	81 (11%)	57 (12%)
Non-Hispanic	631 (88%)	405 (87%)
Not provided (refused)	4 (1%)	3 (1%)
Race		
Caucasian	505 (70%)	319 (69%)
African-American	125 (17%)	90 (19%)
Asian	8 (1%)	5 (1%)
More than one race	9 (1%)	8 (2%)
Other	56 (8%)	39 (8%)
Not Provided (refused)	13 (1%)	4 (1%)
Time Since Injury	7.1 (10.0)	6.56 (9.5)
<1 year post injury	195 (27%)	161 (35%)
1–3 years post injury	186 (26%)	118 (25%)
>3 years post injury	335 (47%)	186 (40%)
Diagnosis		
Paraplegia Complete	182 (25%)	128 (27%)
Paraplegia Incomplete	143 (20%)	90 (19%)
Tetraplegia Complete	157 (22%)	98 (22%)
Tetraplegia Incomplete	230 (32%)	147 (32%)
Unknown/Missing	4 (1%)	2 (0%)

### Preliminary analysis and item removal

Following the first round of analyses on the initial 35-item pool, 2 items were deleted and 5 items were set aside as uncalibrated. Two items related to sleep (“*I had trouble falling asleep*” and “*My sleep was restless*”) were removed. Both of these items exhibited LID, both could be related to a myriad of constructs other than anxiety, and both had been removed from Neuro-QOL during calibration. A subset of 5 items covered aspects of anxiety very specific to SCI (e.g. “*I worried about having a bowel or bladder accident*”, “*I was anxious if my wheelchair/walking aid was not nearby*”) exhibited low item-total correlations (all 5 items) and category inversions (3 items) and as such were removed from the item pool and set aside for possible future use. Repeating analyses of the remaining 31 items, we removed three Neuro-QOL items with slightly modified wording (e.g. “I felt nervous when I *am* left alone” instead of “I felt nervous when I *was* left alone) and two items due to low item-total correlations and local item dependency. One item demonstrated DIF when we calibrated the remaining 26 item set, “*I felt nervous when I was left alone.*” After removing these items, Cronbach's α for final set of 25 items was 0.946; item/total correlations ranged from 0.50 to 0.74.

### Confirmatory factor analysis

We observed unidimensionality (CFI = 0.953; RMSEA = 0.069) and *R*^2^ values exceeding 0.35. No item pairs demonstrated local dependence (i.e. residual correlations >|0.20|) and the ratio of the first to second eigenvalue value was 11.4.

### IRT parameter estimation and model Fit

**Figure 1:**
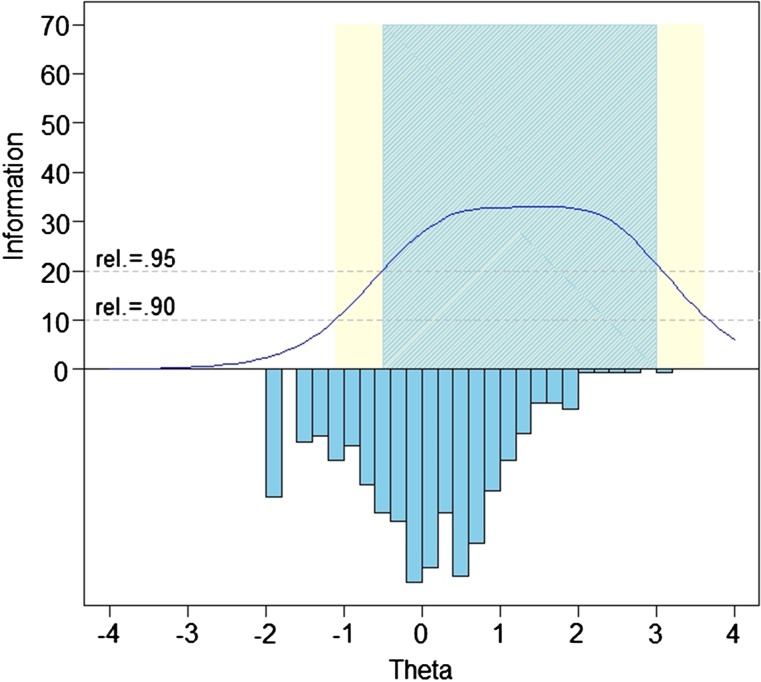
SCI-QOL Anxiety Item Bank Test Information and Precision.

Slopes ranged from 1.17 to 2.52; thresholds ranged from –0.78 to 3.50. Precision in the range between −0.5 and 3.0 theta is equivalent to a classical test theory reliability of 0.95. Fig. 1 shows the bank's test information and precision.

We calculated the S-X^2^ model fit statistics using the IRTFIT^35^ macro program. All items had adequate or better model fit statistics (P > 0.05), with marginal reliability equal to 0.939. No item pairs demonstrated local dependence.

### Differential item functioning

We examined DIF using *lordif*^36^ for six characteristics: age (≤49 vs. ≥50), sex (male *n* = 559 vs. female *n* = 158), education (some college and lower *n* = 523 vs. college degree and above *n* = 194), injury level (tetraplegia *n* = 388 vs. paraplegia *n* = 325), injury severity (incomplete *n* = 374 vs. complete *n* = 339), and time post injury (<1 year *n* = 196 vs. >1 year *n* = 521). Criteria for possible DIF were a statistically significant χ^2^ test (P < 0.01) and effect sizes using McFadden's pseudo *R*^2^ greater than 0.02, a small but non-negligible effect. While we examined 9 items for DIF based on the χ^2^ test, all effect sizes were negligible.

Descriptive statistics for the final 25 items are presented in Table 2.

**Table 2:** SCI-QOL Anxiety Descriptive Item Statistics

Item ID	Item Stem	Mean	SD	% at Min	% at Max
Anxiety_29	I felt trapped in my own body.	2.04	1.262	50.3	6.6
Anxiety_30	I was afraid that I would be unable to live alone.	2.14	1.365	50.1	8.7
EDANX05^PN^	I felt anxious.	2.20	1.134	35.5	4.3
**EDANX07**^PN^	**I felt like I needed help for my anxiety.**	**1.65**	**1.017**	**64.9**	**2.0**
**EDANX18**^PN^	**I had sudden feelings of panic.**	**1.44**	**0.774**	**70.7**	**0.3**
EDANX20^PN^	I was easily startled.	1.70	0.969	57.0	2.1
EDANX26^PN^	I felt fidgety.	1.80	1.033	54.9	2.0
EDANX27^PN^	I felt something awful would happen.	1.63	0.925	60.8	1.4
EDANX30^PN^	I felt worried.	2.22	1.125	35.6	3.6
EDANX33^P^	I felt terrified.	1.36	0.720	75.8	0.4
**EDANX41**^PN^	**My worries overwhelmed me.**	**1.81**	**1.046**	**53.2**	**2.8**
**EDANX46**^PN^	**I felt nervous.**	**2.08**	**1.058**	**39.4**	**2.5**
**EDANX48**^PN^	**Many situations made me worry.**	**2.00**	**1.034**	**41.1**	**2.1**
EDANX51^PN^	I had trouble relaxing.	2.29	1.147	33.0	4.6
**EDANX53**^PN^	**I felt uneasy.**	**2.25**	**1.073**	**31.0**	**2.9**
**EDANX54**^PN^	**I felt tense.**	**2.21**	**1.120**	**35.5**	**3.9**
**EDANX55**^PN^	**I had difficulty calming down.**	**1.56**	**0.835**	**63.4**	**0.7**
NQANX01	I was afraid of what the future holds for me.	2.24	1.216	39.1	5.3
NQANX02^N^	I felt fearful about my future.	2.26	1.223	38.3	5.3
NQANX04^N^	I worried about my physical health.	2.66	1.198	22.5	8.4
**NQANX07**^N^	**I felt nervous when my normal routine was disturbed.**	**1.85**	**1.019**	**50.6**	**1.8**
NQANX17^N^	I suddenly felt scared for no reason.	1.44	0.797	71.4	0.7
NQANX18^N^	I worried about dying.	1.53	.900	68.4	1.3
NQANX19	I was preoccupied with my worries.	1.90	1.033	48.0	1.5
NQANX21	I felt shy.	1.81	0.978	51.4	1.3

^P^ = PROMIS Item.

^N^ = Neuro-QOL Item.

*Context for all items was “In the past 7 days…”.

Response set was 1 = Never/2 = Rarely/3 = Sometimes/4 = Often/5 = Always.

**Bold text** indicates items selected for the short form 9a.

### Transformation to PROMIS metric

We used an algebraic formula to transform Anxiety T-scores to PROMIS’ general population norms such that higher scores indicate more severe anxiety symptoms. Using Stocking-Lord techniques, we calculated transformation constants, slope and intercept, for the 15 “anchor” items (i.e. those items common to both SCI-QOL and PROMIS) and applied these as a linear transformation of each SCI-QOL parameter. Consequently, SCI-QOL Anxiety scores are directly comparable to PROMIS Anxiety scores, with higher scores indicating more severe symptoms of Anxiety. Transformed slopes range from 1.49 to 2.95 and thresholds ranged from −0.91 to 3.38 (see Table 3). The SCI-QOL calibration sample (*n* = 716) mean and standard deviation (SD) were 49.69 (9.60) before transformation and are now 50.75 (9.12). With CAT administration, Assessment Center automatically transforms IRT-based SCI-QOL scores to standard scores on the T metric.

**Table 3:** SCI-QOL Anxiety Item Response Theory Parameters

Item ID	Item Stem	Slope	Threshold 1	Threshold 2	Threshold 3	Threshold 4
Anxiety_29	I felt trapped in my own body.	2.37392	0.13701	0.62937	1.34345	1.83460
Anxiety_30	I was afraid that I would be unable to live alone.	1.49296	0.12077	0.63359	1.32981	2.07949
EDANX05^PN^	I felt anxious.	1.81193	–0.34267	0.51219	1.64793	2.36671
**EDANX07**^PN^	**I felt like I needed help for my anxiety.**	**2.70783**	**0.59202**	**1.03829**	**1.83553**	**2.43617**
**EDANX18**^PN^	**I had sudden feelings of panic.**	**2.60364**	**0.71047**	**1.32843**	**2.36893**	**3.26777**
EDANX20^PN^	I was easily startled.	1.61209	0.36316	1.26653	2.41243	3.01943
EDANX26^PN^	I felt fidgety.	1.88602	0.29583	0.94761	2.01937	2.80279
EDANX27^PN^	I felt something awful would happen.	2.35316	0.44202	1.13056	2.08041	2.66604
EDANX30^PN^	I felt worried.	2.56032	–0.28697	0.34381	1.42237	2.09406
EDANX33^P^	I felt terrified.	2.95125	0.86035	1.47563	2.32914	2.91439
**EDANX41**^PN^	**My worries overwhelmed me.**	**2.69690**	**0.22562**	**0.91617**	**1.68177**	**2.17792**
**EDANX46**^PN^	**I felt nervous.**	**2.22289**	**–0.19025**	**0.52329**	**1.75076**	**2.43653**
**EDANX48**^PN^	**Many situations made me worry.**	**2.74798**	**–0.10969**	**0.65910**	**1.61207**	**2.30627**
EDANX51^PN^	I had trouble relaxing.	2.06440	–0.40607	0.32092	1.46910	2.18751
**EDANX53**^PN^	**I felt uneasy.**	**2.27135**	**–0.47566**	**0.37749**	**1.50217**	**2.33102**
**EDANX54**^PN^	**I felt tense.**	**2.85412**	**–0.27240**	**0.37070**	**1.42042**	**1.99433**
**EDANX55**^PN^	**I had difficulty calming down.**	**2.45540**	**0.51431**	**1.20262**	**2.44249**	**2.95323**
NQANX01	I was afraid of what the future holds for me.	2.23295	–0.20808	0.34944	1.31941	2.00312
NQANX02^N^	I felt fearful about my future.	2.14504	–0.23369	0.34516	1.26921	2.03656
NQANX04^N^	I worried about my physical health.	1.69017	–0.91042	–0.15415	1.22606	2.00613
**NQANX07**^N^	**I felt nervous when my normal routine was disturbed.**	**2.04392**	**0.18734**	**0.90650**	**2.02917**	**2.84207**
NQANX17^N^	I suddenly felt scared for no reason.	2.72940	0.73278	1.36019	2.22805	2.82722
NQANX18^N^	I worried about dying.	1.64426	0.75077	1.47694	2.43390	3.30694
NQANX19	I was preoccupied with my worries.	2.82364	0.08204	0.70816	1.63240	2.44944
NQANX21	I felt shy.	1.58216	0.16933	1.02938	2.36013	3.38166

^P^ = PROMIS Item.

^N^ = Neuro-QOL Item.

*Context for all items was “In the past 7 days…”.

Response set was 1 = Never/2 = Rarely/3 = Sometimes/4 = Often/5 = Always.

**Bold text** indicates items selected for the short form 9a.

### Short form item selection

**Figure 2:**
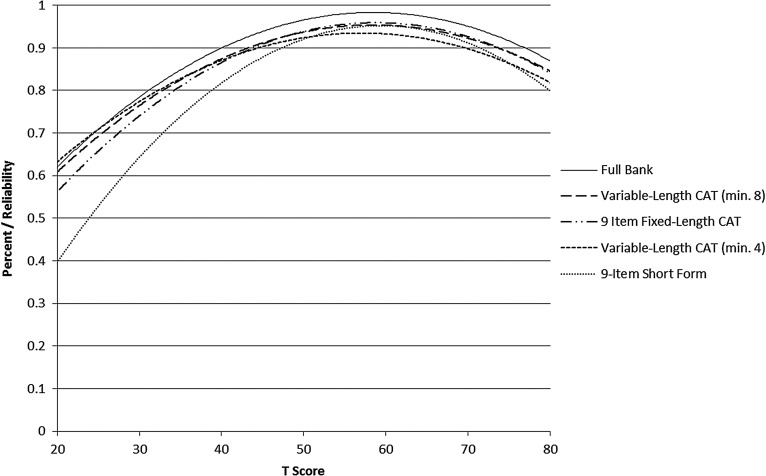
SCI-QOL Anxiety: Measurement Reliability by T-score and Assessment Method.

We developed a 9-item short form of the item bank for situations where it is not feasible to administer items via CAT. We selected items with the greatest information across a wide range of symptom severity. Following PROMIS naming conventions, this form is entitled SCI-QOL Anxiety SF9a. We have compared precision and error of the 25-item bank, 9-item short form, variable-length CAT with a minimum of 4 items, and variable-length CAT with a minimum of 8 items SCI-QOL. Anxiety SF9a raw scores can be converted to IRT-based T-scores using Table 4. Note that participants must complete all 9 SF items to receive a score. Table 5 presents the mean, standard deviation, range, and standard error ranges for the bank, CAT and short form; Fig. 2 shows the associated reliability curves.

**Table 4:** T-score lookup table for SCI-QOL Anxiety Short Form 9a

Raw Score	Scaled (T) Score	Standard Error
9	36.3	5.7
10	41.7	4.1
11	44.1	3.8
12	46.2	3.3
13	47.9	3.1
14	49.4	2.9
15	50.7	2.8
16	51.9	2.7
17	53.0	2.6
18	54.0	2.6
19	55.1	2.6
20	56.0	2.5
21	57.0	2.5
22	57.9	2.5
23	58.9	2.5
24	59.8	2.5
25	60.7	2.5
26	61.6	2.5
27	62.5	2.5
28	63.4	2.5
29	64.3	2.5
30	65.2	2.5
31	66.1	2.5
32	67.0	2.5
33	68.0	2.5
34	68.9	2.5
35	69.8	2.5
36	70.8	2.5
37	71.8	2.5
38	72.8	2.6
39	73.9	2.6
40	75.1	2.7
41	76.4	2.8
42	77.8	2.9
43	79.4	3.1
44	81.3	3.4
45	84.2	3.9

**Table 5:** Breadth of Coverage for SCI-QOL Anxiety and GAD-7

Measure/Mode		# Items Admin			Score					
		Mean(SD)	Min	Max	Mean(SD)	Range	% Floor	% Ceiling	SE	Reliability
**SCI-QOL Anxiety**	Full Bank	25(0)	25	25	50.7(9.1)	31.70–80.50	2.8%	0.1%	0.220	0.948
	9-Item fixed length CAT	9(0)	9	9	50.8(9.0)	33.14–82.23	7.5%	0.1%	0.285	0.921
	Variable-Length CAT (min 4/max 12)	7.2(2.8)	4	12	50.7(9.0)	32.47–82.14	6.8%	0.1%	0.315	0.908
	Variable-Length CAT (min 8/max 12)	8.9(1.6)	8	12	50.8(9.0)	32.47–82.14	6.8%	0.1%	0.286	0.921
	Short Form	9(0)	9	9	50.8(8.6)	36.30–77.50	6.4%	0.1%	0.326	0.899
GAD-7*		7(0)	7	7	3.86(4.34)	0–21	24.9%	0.9%	0.416	0.853

*GAD-7 raw scores; item responses scored 0–3.

### Reliability

For the community-dwelling reliability sample (*n* = 245), the default stopping rules for the CAT were used (minimum of 4 items/maximum of 12 items); the CAT administration averaged 6.67 items (SD 2.8); almost two-thirds (64.0%, *n* = 151) of the sample completed the CAT in 6 items or fewer. Four items were received by 20.3% of the sample, and 15.3% received the maximum number of items (12). The correlation (Pearson's *r*) between the baseline and 1–2 week retest assessments was 0.80 (*n* = 245; P < 0.001), and the ICC (2,1) was 0.80 (95% CI, 0.75 to 0.84).

### GAD-7 crosswalk

**Figure 3:**
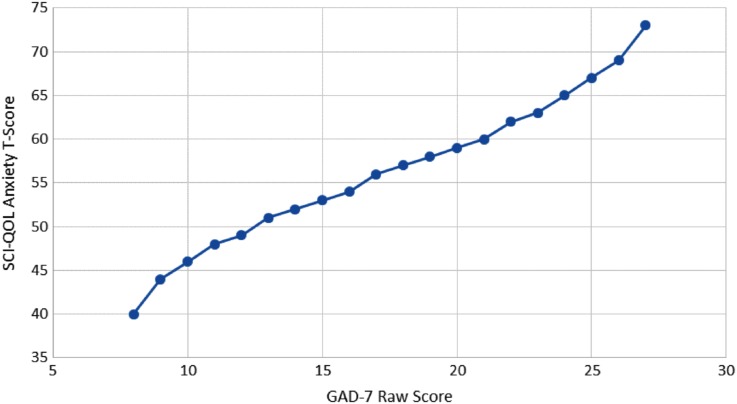
Relationship between SCI-QOL Anxiety and GAD-7 scores. *Individuals reporting no anxiety symptoms (i.e. “never” to all items), and an extremely high amount have been omitted from this graph due to instability of score estimates.

**Figure 4:**
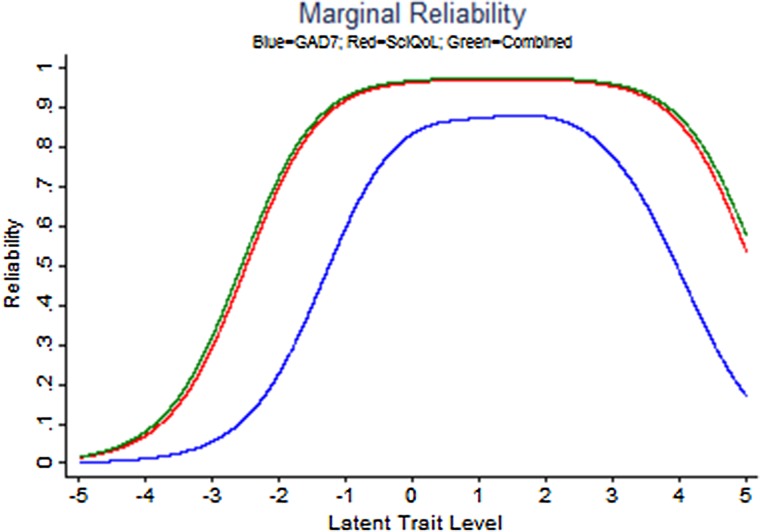
Marginal Reliability of GAD-7, SCI-QOL Anxiety, and Combined (*n* = 465). Colors relate to the online version of the figure.

**Figure 5:**
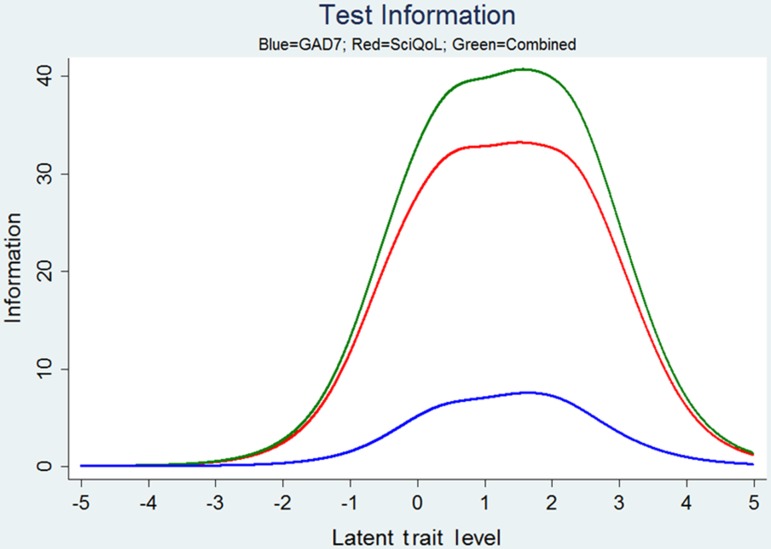
Scale information provided by the GAD-7, SCI-QOL Anxiety, and Combined. Colors relate to the online version of the figure.

We linked the SCI-QOL Anxiety Scale to the GAD-7 using the same procedure used to link the PROMIS Anxiety Scale to the GAD-7.^34^ Fig. 3 displays the relationship between the two measures. The correlation between SCI-QOL Anxiety and GAD-7 was 0.67. Table 6 provides the SCI-QOL Anxiety to GAD-7 conversion; the table allows clinicians and investigators to apply the crosswalk with confidence at a group level because the majority of cases have small differences between the observed and linked mean scores. Fig. 4 shows the marginal reliability at different symptom levels of the SCI-QOL Anxiety, GAD-7, and a score with SCI-QOL and GAD-7 items combined. The SCI-QOL Anxiety items have reliability coefficients above 0.8 for individuals 2 standard deviations below the mean through 4 standard deviations above the mean, much wider range than for the GAD-7. Fig. 5 shows that the test information for the SCI-QOL Anxiety scale is much higher than the GAD-7.

**Table 6:** Linking Table: SCI-QOL Anxiety and GAD-7

**GAD-7** Raw Score	**SCI-QOL** T-Score	**SE**
7	10	9.98
8	40	5.78
9	44	3.9
10	46	2.9
11	48	2.27
12	49	1.86
13	51	1.56
14	52	1.34
15	53	1.17
16	54	1.04
17	56	0.93
18	57	0.85
19	58	0.77
20	59	0.71
21	60	0.66
22	62	0.62
23	63	0.58
24	65	0.55
25	67	0.52
26	69	0.49
27	73	0.47
28	101	0.45

Note: GAD-7 items are scored 1–4 instead of 0–3.

*Individual* level conversion of GAD-7 to SCI-QOL scores should be interpreted cautiously. We estimated an expected SCI-QOL score and then calculated a discrepancy score by subtracting the observed value from the predicted score. For over 40% of the sample, the predicted and observed scores were within a half of a standard deviation, and for over 70% of the sample the predicted score was within 1 SD. However, a substantial number of people (*n* = 131; 28.2%) had discrepancies greater than 1 SD between the observed and predicted scores.

## Discussion

The goals of this study were to develop an item bank to measure anxiety in individuals with SCI, evaluate the bank's psychometric properties, and develop a crosswalk between SCI-QOL Anxiety and the GAD-7. We administered these anxiety items to persons with SCI and developed SCI-specific calibrations using a graded-response IRT model. We removed items demonstrating DIF, local dependence, and poor fit. We linked SCI-QOL Anxiety scores using IRT methods to PROMIS’ general population metric to facilitate score interpretation. As such, the SCI-QOL Anxiety bank is an optimized version of PROMIS V1.0 for individuals with SCI. Clinicians and researchers may specify a desired level of reliability when using CAT administration. Typically, 5 or 6 items are administered in 1 or 2 minutes. Screening requires less reliability; users can specify stopping rules to reduce respondent burden. When greater precision is desired, users can specify more stringent discontinue rule. Short forms can be administered when internet access is unavailable or respondents have difficulty using a computer. Users can be confident that all administration modes yield reliable scores and that an 8-item CAT demonstrates precision nearly equal to the full bank.

Because the GAD-7 is used frequently in biomedical research, we developed a crosswalk to the SCI-QOL Anxiety score. The reliability and test information provided by SCI-QOL Anxiety is superior to the GAD-7 across a much wider range of symptom severity, making SCI-QOL Anxiety preferable, especially when assessing individuals at high and low levels of symptom severity. While the crosswalk is useful at a group level, the correlation of 0.67 between SCI-QOL anxiety and GAD-7 means that score conversions at an individual level are insufficiently precise.

The SCI-QOL Anxiety CAT uses the default criteria used by PROMIS; the minimum number of items to administer is four and the maximum is 12 with a maximum standard error of 0.3. With the default settings, the CAT will administer at least 4 items and discontinue when the standard error of the score estimate is less than 0.3 or 12 items are administered. Users may specify other discontinue criteria to produce more or less precise anxiety estimates. For instance, specifying a minimum of 8 items would result in a lengthier test, but a more reliable score would be obtained.

Scores on the short form are directly comparable to those from CAT or full item bank administration because items are calibrated on the same metric. Short forms may be administered within Assessment Center or may be downloaded for paper administration. Investigators and clinicians can develop custom short forms which can be scored on the same IRT-based metric with the help of a psychometrician.

### Study limitations and future directions

The sample was recruited at 5 SCI Model System sites and one VA medical center. While the sample was stratified to ensure heterogeneity in injury level and severity, it may not be representative of all individuals with SCI living in the United States. The correlation of .67 between SCI-QOL Anxiety and GAD-7 limits the crosswalk to group-level applications. Future studies should evaluate the item bank's utility in clinical applications, use the bank to predict development of anxiety disorders, and evaluate the bank's sensitivity to change. Specifically, it will be important to develop clinically relevant score cut points (or thresholds) and clear scoring interpretation guidelines for clinicians and researchers.

In the future, the SCI-QOL Anxiety CAT or SF could be used clinically at a single time point to screen for elevated anxiety, or could be administered repeatedly over time to identify individuals who are exhibiting increasing levels of anxiety over time. Such information could provide clinicians with a useful starting point for discussion and, potentially, intervention.

## Conclusions

The SCI-QOL Anxiety item bank is a SCI-tailored version of the PROMIS v1.0 Anxiety item bank. It demonstrates a high level of test information across a wide range of symptom severity. An 8-item CAT and 9-item short form demonstrate high levels of reliability. A crosswalk to the GAD-7 provides group-level score conversion.

### Suppliers

*Mplus Statistical Analysis with Latent Variables User's Guide* [computer program]. Version 6. Los Angeles: Muthen & Muthen; 2007.

## Disclaimer statements

**Contributors** All authors have contributed significantly to the design, analysis and writing of this manuscript. The contents represent original work and have not been published elsewhere. No commercial party having a direct financial interest in the results of the research supporting this article has or will confer a benefit upon the authors or upon any organization with which the authors are associated.

**Funding** This study was supported by grant #5R01HD054659 from the National Institutes of Health – co-funded by the Eunice Kennedy Shriver National Institute of Child Health and Human Development/National Center on Medical Rehabilitation Research and the National Institute on Neurological Disorders and Stroke and by grant # U01AR057929 from the National Institutes of Health – an NIH Common Fund Initiative.

**Conflicts of interest** No commercial party having a direct financial interest in the results of the research supporting this article has or will confer a benefit upon the authors or upon any organization with which the authors are associated.

All SCI-QOL items and parameters are © 2015 David Tulsky and Kessler Foundation. All rights reserved. All SCI-QOL items originally from Neuro-QOL are ©2008–2013 David Cella on behalf of the National Institute for Neurological Disorders and Stroke (NINDS). All items are freely available to the public via the Assessment Center platform (www.assessmentcenter.net). There are currently no plans for Dr. Tulsky, Kessler Foundation, or the NINDS to profit from the use of the SCI-QOL instrument.

**Ethics approval** The Institutional Review Board at each site reviewed and approved this project.
